# A genome-annotated bacterial collection of the plant food system microbiota

**DOI:** 10.1128/mra.01221-24

**Published:** 2025-01-14

**Authors:** Laura Pietrantonio, Marion Devers-Lamrani, Peter Thorpe, Catherine Arnton, Senga Robertson-Albertyn, Castrense Savojardo, Alain Hartmann, Fabrice Martin-Laurent, James Abbott, Davide Bulgarelli

**Affiliations:** 1 MS Biotech, Larino, Campobasso, Italy; 2 INRAE, Institut Agro Dijon, Université de Bourgogne, Agroécologie, Dijon, France; 3 Computational Biology, School of Life Sciences, University of Dundee, Dundee, United Kingdom; 4 Plant Sciences, School of Life Sciences, University of Dundee, Dundee, United Kingdom; 5 Department of Pharmacy and Biotechnology, University of Bologna 9296 , Bologna, Italy; SUNY College of Environmental Science and Forestry, Syracuse, New York, USA

**Keywords:** food system microbiota, rhizobia, bacilli, whole-genome sequencing, PacBio HiFi

## Abstract

This study reports draft genomes of 30 bacteria representative of the plant food system microbiota and isolated from different sources in Italy and France. Individual genomes were reconstructed using PacBIO DNA sequencing: taxonomic classification and distribution of genes involved in microbe-environment interactions are reported to facilitate strains' characterization and utilization.

## ANNOUNCEMENT

The microbial communities thriving in association with plants, collectively referred to as the plant microbiota, have long been identified as a resource to reduce the environmental footprint of plant food systems (1). Within the framework of the H2020 Innovation Action “CIRCLES”, we report the sequencing and preliminary characterization of 30 bacterial genomes representative of taxa enriched in the plant food system microbiota and part of collections held at a commercial company in Italy (MS Biotech) and at the Agroecology Department of the French National Research Institute for Agriculture, Food and Environment Bourgogne Franche-Comté (INRAE-BFC).

Cultures of *Rhizobuim sp*., *Bradyrhizobium sp*., and *Rhodococcus sp*. strains were obtained from frozen glycerol stock cultures by inoculating a 10 microliter loop of frozen crystals in liquid broth (TY) (10 g/L tryptone, 5 g/L yeast extract, and 0.06 M CaCl_2_) incubated at 28°C until late exponential phase (from 2 to 5 days). For the other strains described in this study, 50 microliters of glycerol stocks was transferred into 100 mL of Nutrient Broth and incubated at 30°C until exponential phase was reached (~24 hours). Genomic DNA was extracted using the Qiagen Blood & Cell Culture DNA Kit (Cat. No. 19060) and high molecular weight genomic was subsequently purified from lysate using the Qiagen Genomic-tip 500/G columns (Cat. No. 10262) according to manufacturer's instructions. Genomic DNA was quantified by a Qubit fluorometric assay, and its size was estimated by Agilent Tape Station analysis (using the genomic DNA Kit) following the manufacturer's protocol. Sequencing libraries were prepared using the SMRTbell Express Template Prep Kit 2.0 (PacBio, Menlo Park, USA) following manufacturer's recommendations for an insert size target of 10 Kb. Sequencing was performed using the “HiFi sequencing” using the platform PacBio Sequel II (PacBio, Menlo Park, USA) at Genotoul-GeT-PlaGe (Toulouse, France). PacBio HiFi data in fastq format were assembled, including overlap alignment and correction, using hifiasm (version 0.19.5-r587). The final graph assembly was converted to fasta format using gfatools (0.4-r214-dirty). Assemblies were interrogated for quality and contamination using quast (v5.2.0) (2) and Bloobtools (version 1.1.1) (3), respectively. Assembly completeness was assessed using BUSCO (version 5.4.3) (4) with bacteria_odb10 models. Genome annotation was performed using bakta (1.8.1) (5). Genome taxonomic classification was performed using GTDB-tk (version 2.3.2) (6). Orthology, comparative, and phylogenetic analyses were performed using OrthoFinder (version 2.5.5) (7), and bcgTree (version 1.2.0) (8) was used for tree's construction. Annotated genomes were mined using tools in Galaxi Europe (9) and the RAST server (10). We used iTOL (version 6) (11) to visualize and annotate a phylogenetic tree; unless otherwise specified, default parameters were used for the computational tools utilized. Sequencing and assembly information is available in data set “CIRCLES_WP3 S1” accessible at https://zenodo.org/records/14356128 .

**Fig 1:**
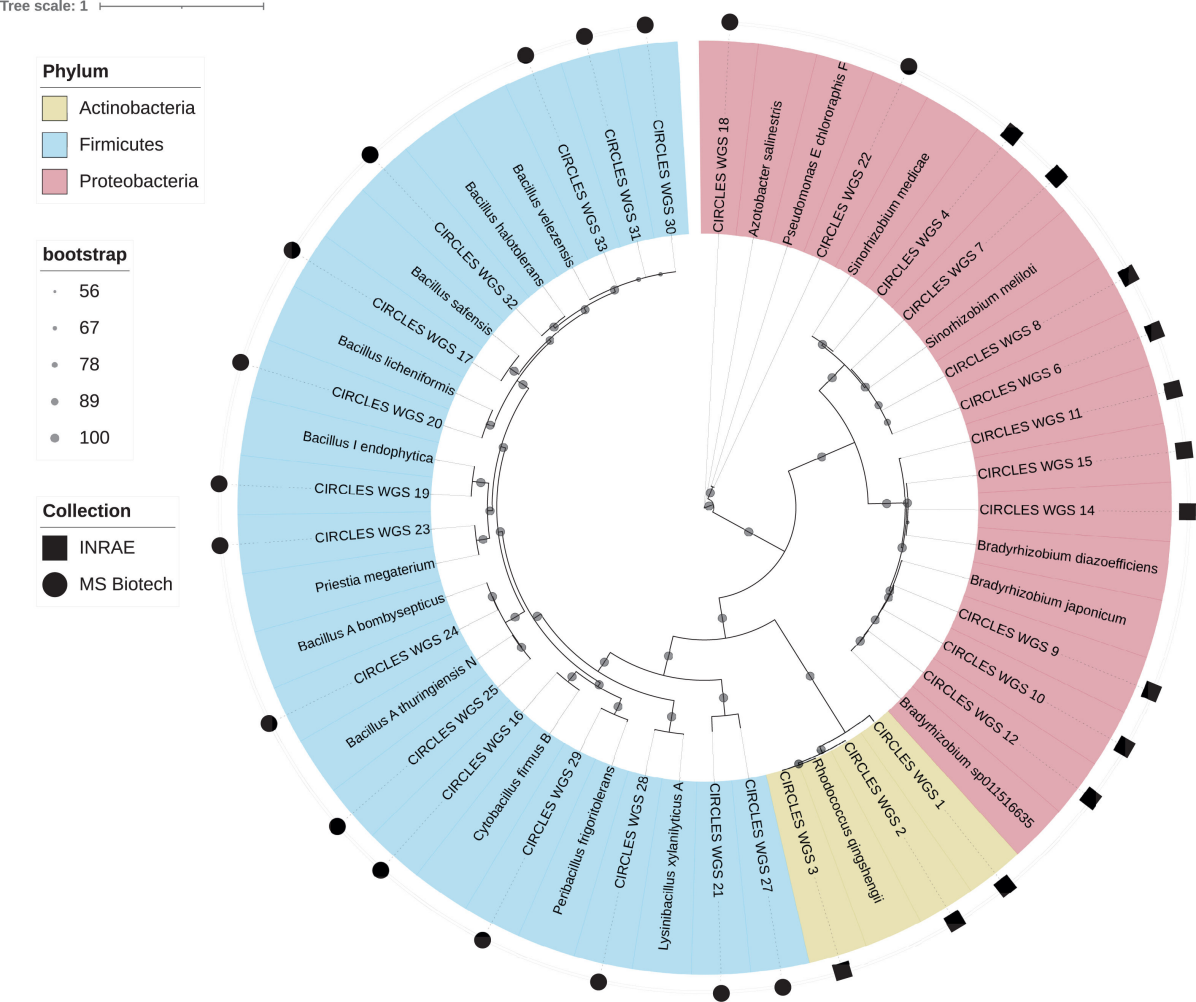
Whole-genome phylogenetic tree of individual genomes (ANI cutoff, 95%) constructed incorporating additional reference sequences, depicted with genus and species name for each clade. The tree was constructed with 100 bootstrap iterations; the color code depicts strain’s taxonomic affiliation at phylum level. The outer shapes indicate strain’s availability in either MS Biotech or INRAE collection.

As a proof of concept, we mined the annotation of individual genomes for key traits in microbe-environment interactions, including genes putatively implicated in (a) nodulation and nitrogen fixation in rhizobia (12), (b) nitrous oxide reduction (13), (c) the production of phytohormones (14), and (d) induced system resistance (15) as summarized in Table 1. To facilitate sample retrieving, we constructed a phylogenetic tree highlighting genomes' relatedness with reference strains (Fig. 1).

**TABLE 1:** Taxonomic affiliation, prediction, functional genes, accession numbers of genomes, and isolation source of the 30 individual bacterial isolates described in this study

Isolate ID and taxonomy^*a*^	*nif*	*nod*	*nosZ*	*cheA*	*YsnE*	*trpA,B*	*men*	*bgl*	Accession	Source & cultivation^*b*^
CIRCLES_WGS1	S,U	-	-	Y	+	+	+	+	SAMEA115779467	Spinach soil, Italy.
*Rhodococcus qingshengii*										Minimal salt medium containing metamitron 100 mg/L as sole source of carbon and nitrogen (16)
CIRCLES_WGS2	U,Z	-	-	Y,C,X	+	+	+	+	SAMEA115779468	
*Rhodococcus qingshengii*										
CIRCLES_WGS3	U	-	-	Y	+	+	+	+	SAMEA115779469	
*Rhodococcus qingshengii*										
CIRCLES_WGS4	U,T,B,A,D,K,E,X,N,H	+	+	A,W,Y,D,B,R,C,X	+	+	+	+	SAMEA115779470	*Trigonella foenum graecum* isolate, France.
*Sinorhizobium medicae*										Reference isolation protocol (17)
CIRCLES_WGS6	T,B,A,D,K,E,X,N,U,S,H	+	+	A,W,Y,D,B,R	+	+	+	+	SAMEA115779471	*Medicago sativa* isolate, France.
*Sinorhizobium meliloti*										Reference isolation protocol (17)
CIRCLES_WGS7	T,B,A,D,K,E,X,N,U,S,H	+	+	A,W,Y,D,B,R,C,X	+	+	+	+	SAMEA115779472	
*Sinorhizobium meliloti*										
CIRCLES_WGS8	T,B,A,D,K,E,X,N,U,S,H	+	-	A,W,Y,D,B,R	+	+	+	+	SAMEA115779473	*Trigonella foenum graecum* isolate, France.
*Sinorhizobium meliloti*										Reference isolation protocol (17)
CIRCLES_WGS9	D,K,E,H,N,X,S,T,B,Q,W,A,U	+	-	A,W,Y,B,R,C,X	+	+	+	+	SAMEA115779474	Soybean nodule isolate, France.
*Bradyrhizobium japonicum*										Reference isolation protocol (17)
CIRCLES_WGS10	D,K,E,H,N,X,S,T,B,Q,W,A,U	+	-	A,W,Y,B,R,C,X	+	+	+	+	SAMEA115779475	
*Bradyrhizobium japonicum*										
CIRCLES_WGS11	D,K,E,H,N,X,S,T,B,Q,W,A,U	+	-	A,W,Y,B.R	+	+	+	+	SAMEA115779476	
*Bradyrhizobium diazoefficiens*										
CIRCLES_WGS12	D,K,E,H,N,X,S,T,B,Q,W,A,U	+	-	W,Y,B.R	+	+	+	+	SAMEA115779477	
*Bradyrhizobium sp011516635*										
CIRCLES_WGS14	D,K,E,H,N,X,S,T,B,Q,W,A,U	+	-	W,Y,B.R	+	+	+	+	SAMEA115779478	
*Bradyrhizobium diazoefficiens*										
CIRCLES_WGS15	D,K,E,H,N,X,S,T,B,Q,W,A,U,Z	+	+	W,Y,B,R	+	+	+	+	SAMEA115779479	
*Bradyrhizobium diazoefficiens*										
CIRCLES_WGS16	S,U	-	-	A,W,Y,D,B,R,C,X	+	+	+	-	SAMEA115779480	Sandy soil, Italy.
*Cytobacillus firmus*										Nutrient agar plus cycloheximide, 30°C
CIRCLES_WGS17	S,U	-	-	A,W,Y,D,B,R,C,X	+	+	+	+	SAMEA115779481	Bean leaf, Italy.
*Bacillus safensis*										Nutrient agar plus cycloheximide, 30°C
CIRCLES_WGS18	F,M,Z,W,V,S,U,X,N,E,Y,T,K,D,H,Q,B,A,L	-	-	A,W,Y,B,R,	+	+	-	-	SAMEA115779482	Wheat rhizosphere, Italy.
*Azotobacter salinestris*										Enrichment culture in nitrogen-free mannitol medium, 25°C (18)
CIRCLES_WGS19	S,U	-	-	A,W,Y,D,B,R	-	+	+	+	SAMEA115779483	Wheat rhizosphere, Italy.
*Priestia endophytica*										Nutrient agar plus cycloheximide, 30°C
CIRCLES_WGS20	S,U	-	-	A,W,Y,D,B,R,C,X	+	+	+	+	SAMEA115779484	Sandy soil, Italy. Peptone-meat extract- KNO_3_ medium, 45°C (19)
*Bacillus licheniformis*										
CIRCLES_WGS21	U	-	-	A,W,Y,D,B,R,C,X	+	+	-	-	SAMEA115779485	Succulent root, Italy.
*Paenibacillus zeisoli*										Nutrient agar plus cycloheximide, 30°C
CIRCLES_WGS22	S,U	-	-	W,Y,B,R	-	+	-	+	SAMEA115779486	Pomegranate roots, Italy
*Pseudomonas chlororaphis*										Triptic soy agar, 30°C
CIRCLES_WGS23	S,U	-	-	A,W,Y,D,B,R	+	+	+	+	SAMEA115779487	*Popillia japonica,* Italy.
*Priestia megaterium*										Nutrient agar plus cycloheximide, 30°C
CIRCLES_WGS24	S,U	-	-	A,W,Y,B,R,	-	+	+	+	SAMEA115779488	Vine leaf treated with copper, Italy.
*Bacillus bombysepticus*										Nutrient agar plus cycloheximide, 30°C
CIRCLES_WGS25	S,U	-	-	A,W,Y,D,B,R	+	+	+	-	SAMEA115779489	Sandy soil, Italy.
*Bacillus thuringiensis*										Nutrient agar plus cycloheximide, 30°C
CIRCLES_WGS27	U,S,X,N,E,K,D,H,B,	-	-	A,W,Y,D,R,C,X	-	+	+	+	SAMEA115779490	*Aloe* leaf compost, Italy.
*Paenibacillus polymyxa*										Nutrient agar plus cycloheximide, 30°C
CIRCLES_WGS28	U	-	-	A,W,Y,D,B,R,C,X	-	+	+	-	SAMEA115779491	Nematode eggs, Italy.
*Lysinibacillus xylanilyticus*										Nutrient agar plus cycloheximide, 30°C
CIRCLES_WGS29	U	-	-	A,W,Y,D,B,R,C,X	+	+	-	+	SAMEA115779492	Underwood, Italy.
*Bacillus simplex*										Nutrient agar plus cycloheximide, 30°C
CIRCLES_WGS30	S	-	-	A,W,Y,D,B,R,C,X	+	+	+	+	SAMEA115779493	Cabbage leaf, Italy.
*Bacillus velezensis*										Nutrient agar plus cycloheximide, 30°C
CIRCLES_WGS31	S	-	-	A,W,Y,D,B,R,C,X	+	+	+	+	SAMEA115779494	Copper-based product MSBiotech, Italy.
*Bacillus velezensis*										Nutrient agar plus cycloheximide, 30°C
CIRCLES_WGS32	S	-	-	A,W,Y,D,B,R,C,X	+	+	+	+	SAMEA115779495	Sandy soil, Italy.
*Bacillus halotolerans*										Nutrient agar plus cycloheximide, 45°C
CIRCLES_WGS33	S	*-*	-	A,W,Y,D,B,R,C,X	+	+	+	+	SAMEA115779496	Blended aloe leaf, Italy.
*Bacillus velezensis*										Nutrient agar plus cycloheximide, 30°C

^*a*^ Strain taxonomy reflects the lowest and unique rank as defined by GTDBTK (v2.3.2).

^*b*^ Unless otherwise specified, incubation lasted for a minimum of 24 hours. Firmicutes were isolated on substrates previously incubated at 80°C for 10 minutes. Numbers (16–19) depict references to isolation protocols.

## Data Availability

The genomic sequences reported in this study are deposited in the European Nucleotide Archive (ENA), project PRJEB75963. Accession numbers for the individual genomes are provided in Table 1. To acquire isolates, or for questions or suggestions, please contact Laura Pietrantonio at MSBiotech or Alain Hartmann and Marion Devers-Lamrani at the Agroecology department of INRAE. The codes to reproduce data analyses are available at https://github.com/bartongroup/PT_HiFi_rhizobial_assemblies/.

## References

[1] Schlaeppi K, Bulgarelli D. 2015. The plant microbiome at work. Mol Plant Microbe Interact 28:212–217. doi:10.1094/MPMI-10-14-0334-FI 25514681

[2] Gurevich A, Saveliev V, Vyahhi N, Tesler G. 2013. QUAST: quality assessment tool for genome assemblies. Bioinformatics 29:1072–1075. doi:10.1093/bioinformatics/btt086 23422339 PMC3624806

[3] Laetsch DR, Blaxter ML. 2017. BlobTools: interrogation of genome assemblies. F1000Res 6:1287. doi:10.12688/f1000research.12232.1

[4] Seppey M, Manni M, Zdobnov EM. 2019. BUSCO: assessing genome assembly and annotation completeness. Methods Mol Biol 1962:227–245. doi:10.1007/978-1-4939-9173-0_14 31020564

[5] Schwengers O, Jelonek L, Dieckmann MA, Beyvers S, Blom J, Goesmann A. 2021. Bakta: rapid and standardized annotation of bacterial genomes via alignment-free sequence identification. Microb Genom 7:000685. doi:10.1099/mgen.0.000685 34739369 PMC8743544

[6] Chaumeil P-A, Mussig AJ, Hugenholtz P, Parks DH. 2020. GTDB-Tk: a toolkit to classify genomes with the Genome Taxonomy Database. Oxford University Press. 10.1093/bioinformatics/btz848 PMC7703759 31730192

[7] Emms DM, Kelly S. 2019. OrthoFinder: phylogenetic orthology inference for comparative genomics. Genome Biol 20:238. doi:10.1186/s13059-019-1832-y 31727128 PMC6857279

[8] Ankenbrand MJ, Keller A. 2016. bcgTree: automatized phylogenetic tree building from bacterial core genomes. Genome 59:783–791. doi:10.1139/gen-2015-0175 27603265

[9] Galaxy Community. 2022. The Galaxy platform for accessible, reproducible and collaborative biomedical analyses: 2022 update. Nucleic Acids Res 50:W345–W351. doi:10.1093/nar/gkac247 35446428 PMC9252830

[10] Aziz RK, Bartels D, Best AA, DeJongh M, Disz T, Edwards RA, Formsma K, Gerdes S, Glass EM, Kubal M, et al.. 2008. The RAST Server: rapid annotations using subsystems technology. BMC Genomics 9:1–15. doi:10.1186/1471-2164-9-75 18261238 PMC2265698

[11] Letunic I, Bork P. 2024. Interactive tree of life (iTOL) v6: recent updates to the phylogenetic tree display and annotation tool. Nucleic Acids Res 52:W78–W82. doi:10.1093/nar/gkae268 38613393 PMC11223838

[12] Masson-Boivin C, Sachs JL. 2018. Symbiotic nitrogen fixation by rhizobia-the roots of a success story. Curr Opin Plant Biol 44:7–15. doi:10.1016/j.pbi.2017.12.001 29289792

[13] Stein LY. 2020. The long-term relationship between microbial metabolism and greenhouse gases. Trends Microbiol 28:500–511. doi:10.1016/j.tim.2020.01.006 32396828

[14] Spaepen S, Vanderleyden J, Remans R. 2007. Indole-3-acetic acid in microbial and microorganism-plant signaling. FEMS Microbiol Rev 31:425–448. doi:10.1111/j.1574-6976.2007.00072.x 17509086

[15] Pieterse CMJ, Zamioudis C, Berendsen RL, Weller DM, Van Wees SCM, Bakker PAHM. 2014. Induced systemic resistance by beneficial microbes. Annu Rev Phytopathol 52:347–375. doi:10.1146/annurev-phyto-082712-102340 24906124

[16] Billet L, Devers M, Rouard N, Martin-Laurent F, Spor A. 2019. Labour sharing promotes coexistence in atrazine degrading bacterial communities. Sci Rep 9:18363. doi:10.1038/s41598-019-54978-2 31798012 PMC6892810

[17] Vincent J. 1970. A manual for practical study of root nodules bacteria. IBP. Handbook No. 15. Black Well Sci. Publications. Oxford and Ed. inburg.

[18] Jiménez DJ, Montaña JS, Martínez MM. 2011. Characterization of free nitrogen fixing bacteria of the genus Azotobacter in organic vegetable-grown Colombian soils. Braz J Microbiol 42:846–858. doi:10.1590/S1517-83822011000300003 24031700 PMC3768769

[19] Leitzmann C, Bernlohr RW. 1965. Changes in the nucleotide pool of Bacillus licheniformis during sporulation. J Bacteriol 89:1506–1510. doi:10.1128/jb.89.6.1506-1510.1965 14291588 PMC277684

